# Differential side-effects of *Bacillus thuringiensis* bioinsecticide on non-target *Drosophila* flies

**DOI:** 10.1038/s41598-020-73145-6

**Published:** 2020-10-01

**Authors:** Aurélie Babin, Marie-Paule Nawrot-Esposito, Armel Gallet, Jean-Luc Gatti, Marylène Poirié

**Affiliations:** Institut Sophia Agrobiotech, Université Côte D’Azur, INRAE, CNRS, ISA, 400 route des chappes, 06903 Sophia Antipolis, France

**Keywords:** Developmental biology, Ecology, Ecology

## Abstract

Bioinsecticides based on *Bacillus thuringiensis* (*Bt*) spores and toxins are increasingly popular alternative solutions to control insect pests, with potential impact of their accumulation in the environment on non-target organisms*.* Here, we tested the effects of chronic exposure to commercial *Bt* formulations (*Bt* var. *kurstaki* and *israelensis*) on eight non-target *Drosophila* species present in *Bt*-treated areas, including *D. melanogaster* (four strains)*.* Doses up to those recommended for field application (~ 10^6^ Colony Forming Unit (CFU)/g fly medium) did not impact fly development, while no fly emerged at ≥ 1000-fold this dose. Doses between 10- to 100-fold the recommended one increased developmental time and decreased adult emergence rates in a dose-dependent manner, with species-and strain-specific effect amplitudes. Focusing on *D. melanogaster*, development alterations were due to instar-dependent larval mortality, and the longevity and offspring number of adult flies exposed to bioinsecticide throughout their development were moderately influenced. Our data also suggest a synergy between the formulation compounds (spores, cleaved toxins, additives) might induce the bioinsecticide effects on larval development. Although recommended doses had no impact on non-target *Drosophila* species, misuse or local environmental accumulation of *Bt* bioinsecticides could have side-effects on fly populations with potential implications for their associated communities.

## Introduction

The world's population is expected to reach more than 9.7 billion people by 2050^1^, increasing the demand for food. This requires fighting pests, especially insect pests that cause more than 30% of agricultural losses^2^. Nowadays, their management relies heavily on chemical insecticides. However, their use starts to be reduced due to the emergence of resistance, the appearance of secondary pests, the adverse side-effects on non-target species (pests’ natural enemies, pollinators)^3,4^, and importantly the impacts on human health and biodiversity^5,6^. Developed as more specific and safer alternatives, bioinsecticides represent 5% of the pesticide market, the large majority being microbial insecticide formulations based on viable spores and toxins of the bacterium *Bacillus thuringiensis* (*Bt*) (over 400 registered formulations)^4,7^.

*Bt* is a Gram-positive endospore-forming bacterium that synthesizes a wide range of toxins with different chemical structures, modes of action and biological targets. The most abundant and studied are Cry δ-endotoxins encoded by genes located on large plasmids and produced as parasporal crystalline inclusions during the stationary growth phase^8,9^. *Bt* produces other insecticidal toxins, the Cyt (cytolytic δ-endotoxins) and Vip (secreted Vegetative Insecticidal Proteins) that synergize their effects with Cry toxins, virulence factors such as β-exotoxins (or thuringiensin), a secreted nucleotide toxic for almost all tested life forms thus prohibited in commercial formulations^10^, and anti-fungal factors^11^. *Bt* subspecies and strains can differ in their plasmid number and in the synthesized toxins cocktail responsible for their biological activity, which determine potential target insects^12^. For instance, *Bt* var. *kurstaki* (*Btk*), used mainly against lepidopteran larvae, produces 5 Cry toxins (Cry1Aa, Cry1Ab, Cry1Ac, Cry2Aa and Cry2Ab)^13^, while *Bt* var. *israelensis* (*Bti*), used mainly against mosquitoes and black flies, produces a combination of Cry4Aa, Cry4Ba, Cry10Aa, and Cry11Aa^14^. The different toxin cocktails produced by some *Bt* subspecies can also be detrimental to non-insect organisms such as nematodes, protozoa, and even molluscs^12^.

The bioinsecticide formulations based on spores and toxin crystals of *Btk* and *Bti* are the most sprayed in organic and conventional farming, and in natural areas (e.g. forests, swamps). It is generally accepted that once ingested by insect larvae, the toxin crystals are dissolved by the midgut alkaline pH, releasing ~ 130 kDa pro-toxins that are then processed by digestive proteases into smaller, soluble, active toxin fragments of ~ 60–70 kDa^15,16^. Active toxins bind to specific receptors of midgut epithelial cells, eliciting pores formation in the cell membrane, cell lysis and gut epithelium disorganization^17^. This allows gut bacteria, including *Bt*, to colonize the hemocoel, and leads to rapid septicaemia and death^18^.

Numerous impact studies of field application rates and acute intoxications have shown that *Bt* bioinsecticides are safe or have a limited impact on non-target vertebrates and invertebrates, and associated species communities^19,20^. However, the increasing use of bioinsecticides based on *Bt* spores and toxins has recently raised concern^21^ and led to the assessment of their potential effects on non-target species, such as auxiliary insects of biological control^22^, pollinators^23^ and species communities which share their habitat with *Bt*-targeted insect pests^24–26^. There is growing evidence of direct and indirect cross-effects of *Bt* bioinsecticide formulations and *Bt*- Cry and Cyt toxins across insect species and orders, or even across phyla, suggesting that *Bt* targeting is only partly specific^12,26,27^. Data also showed that almost all of the applied *Btk* formulation dose was still present on the leaves surface 72 h after spraying^28^, its amount returning close to environmental levels only 28 days after treatment^29^. Finally, *Bt* spores can survive in the soil and on different supports for months and even years after application^30–33^. *Bt* formulations contain also different compounds to protect spores and crystals and aggregate them into a wettable form, surfactants to facilitate spraying and dispersion on plants, and phagostimulants^34,35^. Nevertheless, since toxin crystals, and to a much lesser extent spores^36^, are somewhat sensitive to abiotic conditions (e.g. UV, pH, rainfall), repeated spraying with a minimum delay of 3 to 8 days is often recommended over the period of pest occurrence to achieve the required pest control level^35,37^ (https://www.certiseurope.fr; https://www.certisusa.com). All these can potentially lead to *Bt* accumulation in the environment, thus raising the rarely addressed issue of potential side-effects of chronic exposure (i.e. continuous and increasing exposure dose for an extended period) of non-target species to doses unexpectedly above those that are recommended.

Diptera are worldwide distributed insects, most of which are not targets of commercial *Bt* formulations. This is the case of the genus *Drosophila*, represented by ~ 1500 described species^38^, including the model organism *Drosophila melanogaster*. In the field, most of these flies feed and reproduce mainly on ripening or rotting/fermenting fruits and are therefore naturally present in areas treated with *Bt* such as orchards, vineyards and gardening areas. Unable to disperse between food patches, early developmental stages of *Drosophila* eat intensively and grow exponentially^39^ and may thus ingest high doses of *Bt* bioinsecticides that have accumulated during the treatment periods. Surprisingly, despite the presence of many *Drosophila* species in *Bt-*treated areas, their role in the decomposition of organic matter, and the ease of study of some species, only a few studies have focused on these flies. However, most of them suggested susceptibility to *Btk*, but they used mainly late 3rd instar larvae preparing for pupation, which do not feed much. In addition, these studies used *Bt* preparations, especially field isolates, that possibly contained highly toxic β-exotoxins, which are not authorized in commercial *Bt* formulations^40–47^. So far, no study addressed the effects of chronic exposure to commercial *Bt* formulations on developing stages of these Dipterans that are present in *Bt*-treated areas.

Here, we tested the chronic side-effects of commercial formulations of *Btk* and, to a lesser extent of *Bti*, on non-target *Drosophila* flies (*D. melanogaster* and seven other *Drosophila* species), with doses starting from mean recommended spray doses up to ~ 1000 times this dose (i.e. below acute intoxication doses used in most studies). We mainly focused on developmental traits (developmental time, emergence rate), but also on two fitness-related traits (longevity and offspring number) of adult flies that developed from the egg under *Btk* formulation exposure. Our study would be a first-step in the exploration of potential implications of chronic exposure to *Btk* formulation on *Drosophila* flies.

## Results

### *Btk *formulations adversely impact the development of *D. melanogaster*

**Figure 1:**
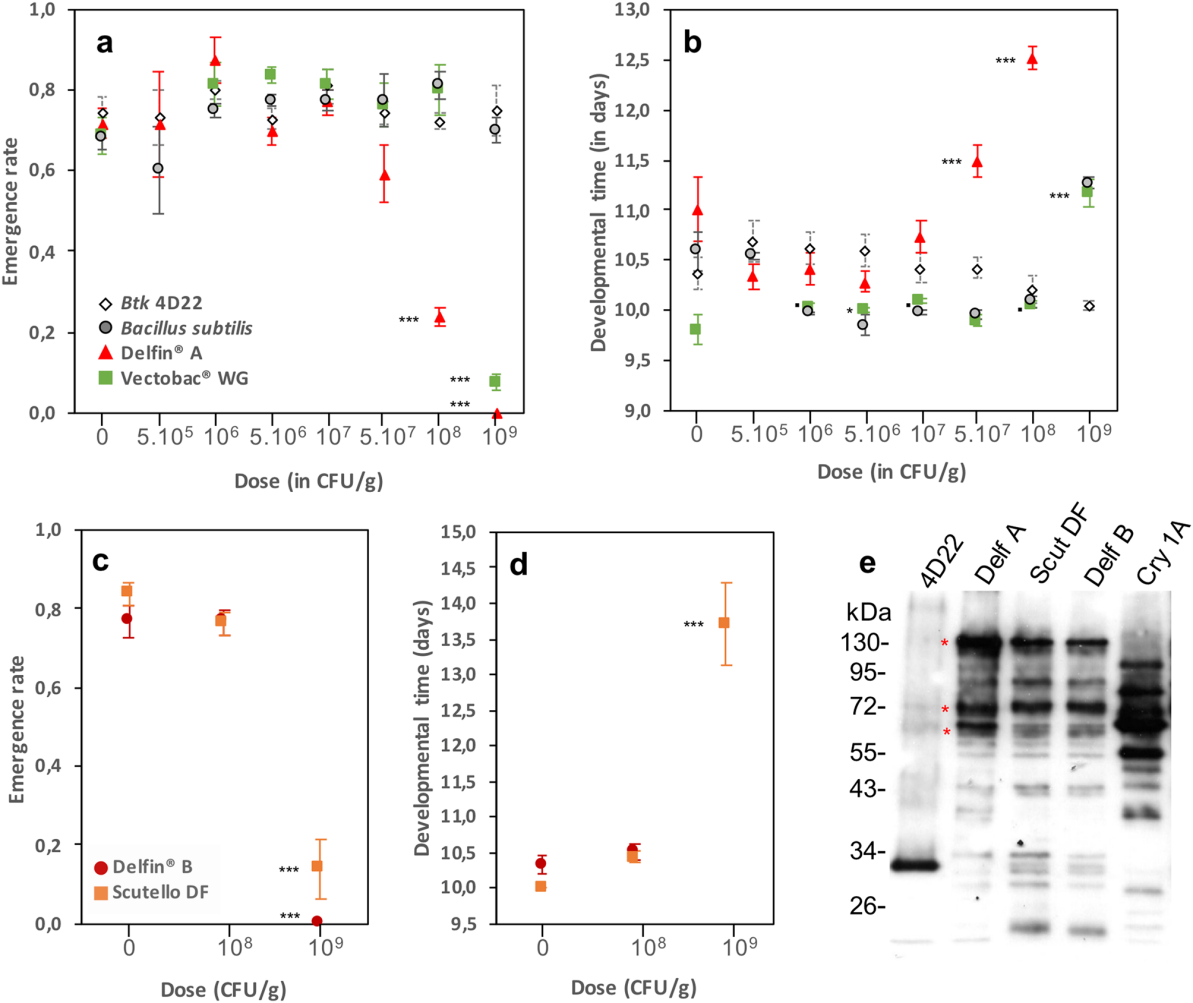
Development of *D. melanogaster* Canton S flies on *Btk* and *Bti* commercial formulations. (**a**) Emergence rate and (**b**) developmental time (mean ± s.e.m.) of 20 initial eggs on increasing doses of *Btk* **DELFIN** A (red triangles), the Cry-free *Btk* 4D22 (open lozenges), the mosquito-targeting *Bti* **VectoBac WG** (green squares) and the non-pathogenic *Bacillus subtilis* (light grey circles). For **VectoBac WG** and *B. subtilis*, *N* = 4–7 per dose; for **DELFIN** A and *Btk* 4D22, *N* = 9–12 for the control, *N* = 3 for 5.10^5^ and 10^9^, *N* = 4–9 for 10^6^, *N* = 7–14 from 5.10^6^ to 10^8^. (**c**) Emergence rate (mean ± s.e.m.) and (**d**) developmental time (mean ± s.e.m.) on increasing doses of the two *Btk* formulations **DELFIN** B (dark red circles) and **Scutello DF** (orange squares). *N* = 4 replicates of 20 eggs per dose and formulation, except for controls and 10^8^ CFU/g of **DELFIN** B (9–10 replicates of 20 eggs). Results of post hoc comparisons of each dose to the control: ^•^0.05 < *P* < 0.1; *0.01 < *P* < 0.05; **0.001 < *P* < 0.01; ***P < 0.001. (**e**) Immunoblotting with an anti-Cry1A polyclonal antibody on proteins from a suspension of laboratory-produced spores of Cry-free *Btk* 4D22, the three *Btk* formulations **DELFIN** A, B, **Scutello DF** and a suspension of laboratory-produced Cry1A toxins. Red asterisks indicate the Cry protoxins (~ 130 kDa) and the activated fragments (~ 60 kDa and ~ 70 kDa).

In a dose–response assay, emergence rates (ER) and developmental times (DT) of wild-type *D. melanogaster* Canton S flies exposed to doses up to 10^7^ CFU/g of **DELFIN** A in a standard low-protein/high-sugar fly medium were similar to those of the control unexposed group (Fig. 1a,b; Table 1). At higher doses, both ER and DT were affected in a dose-dependent manner: ER was reduced by 17% at 5 × 10^7^ CFU/g (although not statistically significant), up to 100% at 10^9^ CFU/g, at which no individual reached the pupal stage. The lethal dose 50 (LD50) was estimated between 5 × 10^7^ and 10^8^ CFU/g (Fig. 1a). DT was increased of about 0.5 day at 5 × 10^7^ CFU/g (+ 4% versus controls) to up to 1.5 days (+ 14%) at 10^8^ CFU/g (Fig. 1b; Table 1). The sex-ratio at emergence (SR, proportion of males) was strongly biased towards males at 10^8^ CFU/g, with 58% more males compared to the control (Supplementary information S2).

**Table 1:** Results of statistical analyses to assess the effect of the dose of formulation/spore production and its interaction with the treatment, the larval instar, the experiment, the sex, the fly strain and the fly species when appropriate.

Source of variation/data	χ^2^/deviance	d.f	*P* value
**Development on Btk DELFIN A, Btk 4D22, Bti VectoBac WG, Bacillus subtilis**			
*Emergence rate*			
Dose × treatment	285.7	20	** < 0.0001**
Dose for each treatment:			
** - DELFIN** A	237.5	6	** < 0.0001**
- 4D22	7.0	7	0.40
** - VectoBac WG**	165.8	5	** < 0.0001**
* - B. subtilis*	1.9	6	0.93
*Developmental time*			
Dose × treatment	220.8	19	** < 0.0001**
Dose for each treatment:			
** - DELFIN A**	68.8	6	** < 0.0001**
- 4D22	16.08	7	**0.024**
** - VectoBac WG**	37.5	6	** < 0.0001**
* - B. subtilis*	13.5	7	0.060
**Development on Btk DELFIN B and Scutello DF (dose effect)**			
*Emergence rate*			
** - DELFIN** B	151.2	2	** < 0.0001**
** - Scutello DF**	105.1	2	** < 0.0001**
*Developmental time*			
** - DELFIN** B	2.5	1	0.12
** - Scutello DF**	30.9	2	** < 0.0001**
**Role of formulation components in the development alterations (dialysis)**			
Dose effect:			
Emergence rate	459.8	3	** < 0.0001**
Developmental time	13.7	2	**0.0011**
**Survival of larval stages on DELFIN A**			
*Cumulative survival*			
Dose × larval instar	16.2	5	**0.0063**
Dose for each instar:			
- Late 1st instar	87.4	5	** < 0.0001**
- Late 2nd instar	25.7	5	**0.0001**
*24-h survival*			
Dose × larval instar	15.9	5	**0.007**
Dose for each instar:			
- Late 1st instar	55.9	5	** < 0.0001**
- Late 2nd instar	3.76	5	0.58
**Adult fitness-related traits after development on DELFIN A**			
*Longevity*			
Experiment	20.1	1	** < 0.0001**
- 1st experiment			
Dose	12.3	3	**0.0065**
Sex (e^β^ coefficient males vs. females ± se: 0.55 ± 0.16)	35.0	1	** < 0.0001**
Dose × sex	20.4	3	**0.00014**
Sexes analyzed separately			
Females (e^β^ coefficients vs. control ± se: 5 × 10^6^: 1.05 ± 0.17, 5 × 10^7^: 0.71 ± 0.16, 10^8^: 0.60 ± 0.21)	12.0	3	**0.0073**
Males (e^β^ coefficients vs. control ± se: 5 × 10^6^: 0.80 ± 0.16, 5 × 10^7^: 0.66 ± 0.16, 10^8^: 1.53 ± 0.18)	20.4	3	**0.00014**
**Adult fitness-related traits after development on DELFIN A**			
- 2nd experiment			
Dose	16.5	3	**0.00090**
Sex (e^β^ coefficient males vs. females ± se: 0.45 ± 0.22)(e^β^ coefficient males vs. females ± se: 0.45 ± 0.22)	31.5	1	** < 0.0001**
Dose × sex	0.69	3	0.88
Sexes analyzed separately			
Females (e^β^ coefficients doses vs. control ± se: 5 × 10^6^: 0.92 ± 0.22, 5 × 10^7^: 0.63 ± 0.21, 10^8^: 0.51 ± 0.21)	13.2	3	**0.0043**
Males (e^β^ coefficients doses vs. control ± se: 5 × 10^6^: 1.02 ± 0.22, 5 × 10^7^: 0.70 ± 0.22, 10^8^: 0.64 ± 0.22)	7.01	3	**0.072**
*Total numbers of offspring*			
Dose × experiment	28.1	3	** < 0.0001**
Dose for each experiment:			
- 1st experiment	26.3	3	** < 0.0001**
- 2nd experiment	4.1	3	0.25
**Development of other strains of D. melanogaster on DELFIN A (including Canton S)**			
*Emergence rate*			
Dose × fly strain	105.5	15	** < 0.0001**
Dose for each fly strain:			
- Canton S	588.6	5	** < 0.0001**
- Nasrallah	745.3	5	** < 0.0001**
- Sefra	900.7	5	** < 0.0001**
- YW1118	636.9	5	** < 0.0001**
*Developmental time*			
Dose × fly strain	9.3	12	0.68
Dose for each fly strain:			
- Canton S	40.3	4	** < 0.0001**
- Nasrallah	18.0	4	**0.0012**
- Sefra	27.2	4	** < 0.0001**
- YW1118	28.9	4	** < 0.0001**
**Development of other Drosophila species on DELFIN A**			
*Emergence rate*			
Dose × fly species	538.2	30	** < 0.0001**
Dose for each species:			
- *D. simulans*	461.0	5	** < 0.0001**
- *D. yakuba*	750.7	5	** < 0.0001**
*- D. hydei*	596.8	5	** < 0.0001**
- *D. immigrans*	726.3	5	** < 0.0001**
*- D. subobscura*	729.6	5	** < 0.0001**
*- D. suzukii*	725.0	5	** < 0.0001**
- *D. busckii*	586.0	5	** < 0.0001**
*Developmental time*			
Dose × fly species	59.9	22	** < 0.0001**
Dose for each species:			
- *D. simulans*	25.9	4	** < 0.0001**
*- D. yakuba*	34.7	4	** < 0.0001**
- *D. hydei*	11.5	4	**0.022**
*- D. immigrans*	6.01	3	0.11
- *D. subobscura*	68.8	4	** < 0.0001**
*- D. suzukii*	11.7	3	**0.0085**
- *D. busckii*	58.8	4	** < 0.0001**

See figures for post hoc comparisons of the doses with the control dose.

Significant statistical differences are indicated in bold

We observed no change in ER using the same dose range of the *Btk* Cry-free strain 4D22 (Fig. 1a,e; Table 1) and the non-pathogenic *Bacillus subtilis* (Fig. 1a, Table 1), two controls for the effect of ingestion of high loads of spores. In contrast, addition of the formulation of *Bt var. israelensis* **VectoBac WG** reduced ER by 89% only at 10^9^ CFU/g (~ 2000 times the recommended dose; Fig. 1a; Table 1; Supplementary information S1). DT varied with the dose of *Btk* 4D22, the differences being mainly between doses but not with the control. DT increased by ~ 1.5 days at the highest dose of **VectoBac WG** (Fig. 1b; Table 1) and showed a similar trend with *B. subtilis* (*P* = 0.06; Fig. 1b; Table 1). None of these treatments influenced dramatically the SR (Supplementary information S2).

To test whether these effects are generic to *Btk* formulations, the fly development was evaluated on two other formulations, **DELFIN** B (same brand) and **Scutello DF** (brand Dipel), at the critical doses 10^8^ and 10^9^ CFU/g. As **DELFIN** A, these formulations contain spores and Cry toxins such as Cry-1A as pro-toxins of ~ 130 kDa, activated toxins of ~ 60–70 kDa, but also as smaller fragments^20^ (Fig. 1e, red asterisks). ER remained unchanged at 10^8^ CFU/g whereas no individual reached pupation at 10^9^ CFU/g on **DELFIN** B and very few individuals reached the adult stage on **Scutello DF**, DT being increased by more than 2 days (Fig. 1c,d; Table 1). No significant bias in SR was observed for either formulation (Supplementary information S2).

### *Btk* formulation strongly impacts survival during the larval stages

**Figure 2:**
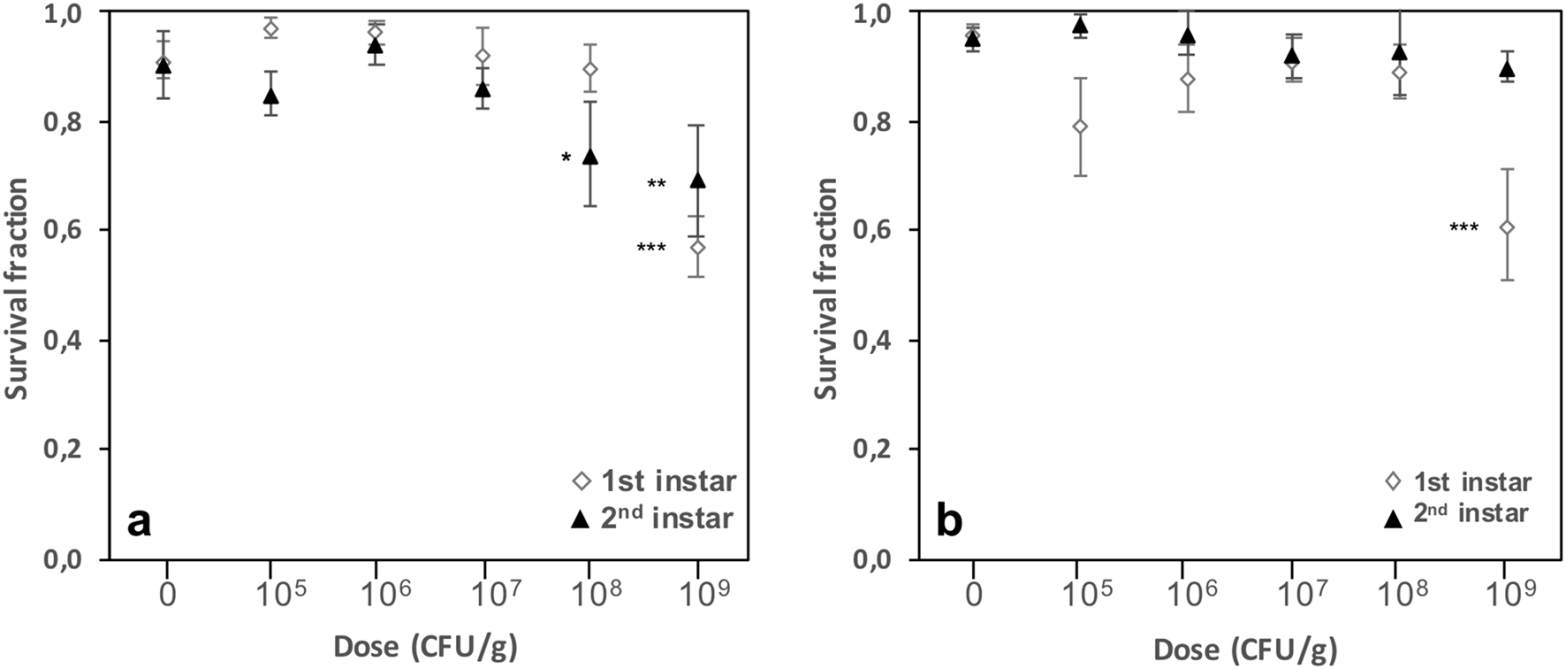
Survival of *D. melanogaster* Canton S larval stages on increasing doses of *Btk* **DELFIN** A. (**a**) Proportion of surviving larvae (mean ± s.e.m.) upon *Btk* formulation exposure from the egg to late 1st instar (open lozenges) and late 2nd instar (black triangles). (**b**) Proportion of surviving larvae (mean ± s.e.m.) upon 24-h *Btk* formulation exposure of early 1st instar larvae (open lozenges) and 2nd instar larvae (black triangles). *N* = 5–7 replicates of 20 individuals per dose. Results of post hoc comparisons of each dose with the control: *0.01 < *P* < 0.05; **0.001 < *P* < 0.01; ***P < 0.001.

Cumulative exposure to **DELFIN** A from the egg to late stages of the 1st and 2nd instars did not influence larval survival at 10^7^ CFU/g but reduced it for both instar larvae above this dose to reach up to 37% mortality at 10^9^ CFU/g (Fig. 2a). Reduced survival tended to occur at a lower dose when cumulative survival was measured later in the development, *i.e.* 10^9^ CFU/g for late 1st instar larvae and 10^8^ CFU/g for late 2nd instar larvae (Fig. 2a; Table 1). For both instars, larvae surviving at 10^9^ CFU/g were noticeably smaller and less active than those surviving at lower doses, and none of these individuals reached the pupal stage (see results above). A 24-h exposure of the 1st or 2nd instar larvae resulted in a 36% decrease in survival of 1st instar larvae at 10^9^ CFU/g, whereas survival of 2nd instar larvae was unchanged (Fig. 2b, Table 1).

### Developmental exposure to *Btk* formulation does not strongly influence fitness-related traits in adults

**Figure 3:**
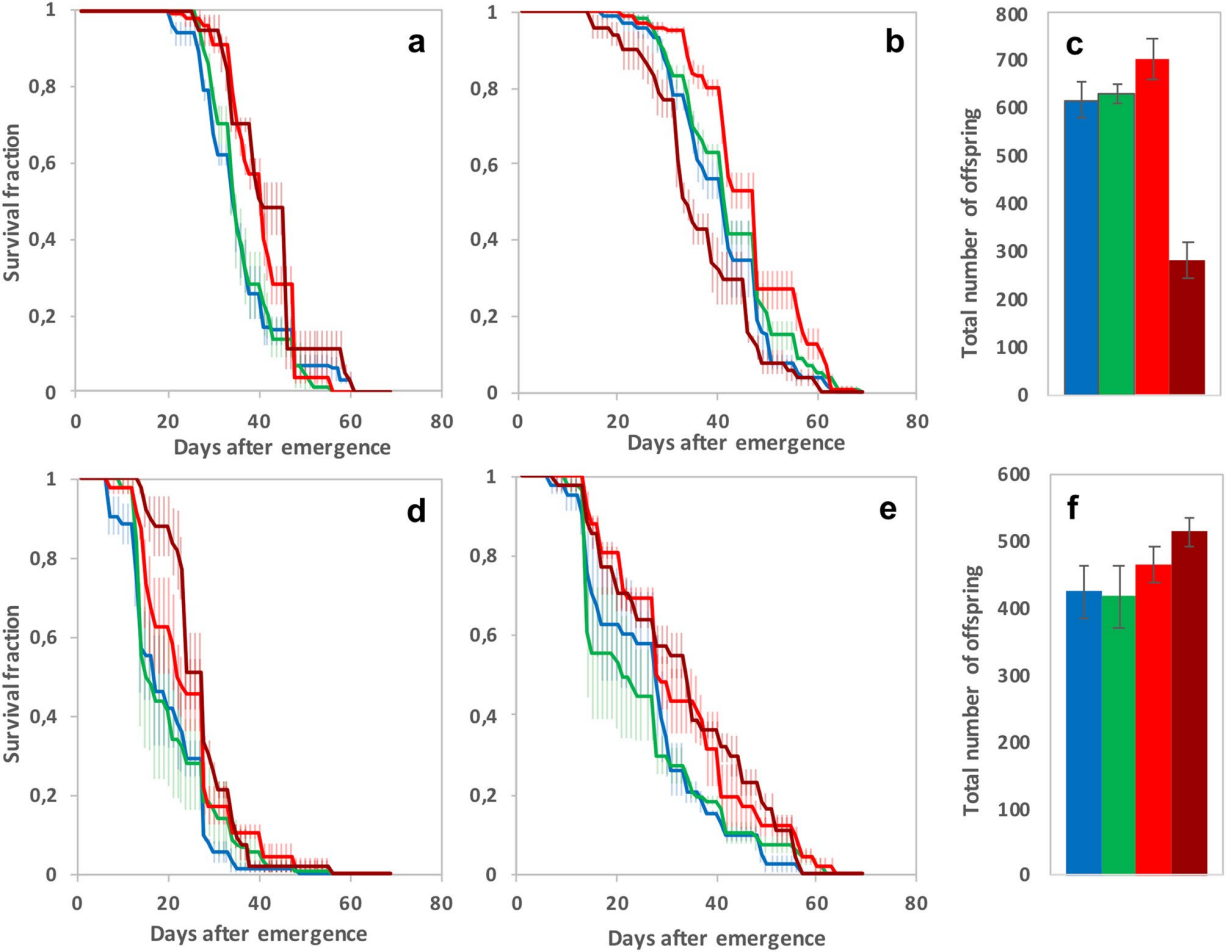
Fitness-related traits of adults (longevity and total offspring number) after development on *Btk* **DELFIN** A. (**a**,**d**) Female longevity (mean survival fraction over time ± s.e.m.), (**b**,**e**) Male longevity (mean ± s.e.m.), and (**c**,**f**) total offspring number (mean ± s.e.m.), measured on individuals that developed without *Btk* formulation (blue items) and on 5 × 10^6^ CFU/g of *Btk* **DELFIN** A (green items), 5 × 10^7^ CFU/g (red items), and 10^8^ CFU/g (dark red items). Data from 2 experiments (**a**–**c**, experiment 1; **d**–**f**, experiment 2). For each trait, *N* = 3–5 replicates of 15 males and 15 females per dose in experiment 1, *N* = 3 replicates of 15 males and 15 females in experiment 2. Results of post hoc comparisons of each dose with the control: *0.01 < *P* < 0.05; **0.001 < *P* < 0.01; ***P < 0.001.

Despite a large variation between the two independent sets of experimental replicates (Table 1), the longevity of adults reared on 5 × 10^6^ CFU/g of **DELFIN** A in low-protein/high-sugar medium was similar to that of non-exposed controls (Fig. 3). Males and females which developed on the two highest doses showed a moderate longevity benefit, higher in females for 10^8^ CFU/g (Fig. 3a,b,d,e; Table 1). Males generally survived better than females (Table 1) but their longevity benefit of developing on 10^8^ CFU/g was only observed in one experiment (Fig. 3b,e).

The number of offspring produced by the 15 females of each fly group during the longevity experiment varied depending on both the experiment and the **DELFIN** A dose (Table 1). In the 1st experiment, adults from larvae reared on 10^8^ CFU/g had fewer offspring compared to the controls and to adults developed on the other doses whereas the total offspring number varied regardless of the **DELFIN** A dose in the 2nd experiment (Fig. 3c,f, Table 1).

### Developmental alterations dependent on *Btk* formulation dose are not specific to *D. melanogaster* Canton S

**Figure 4:**
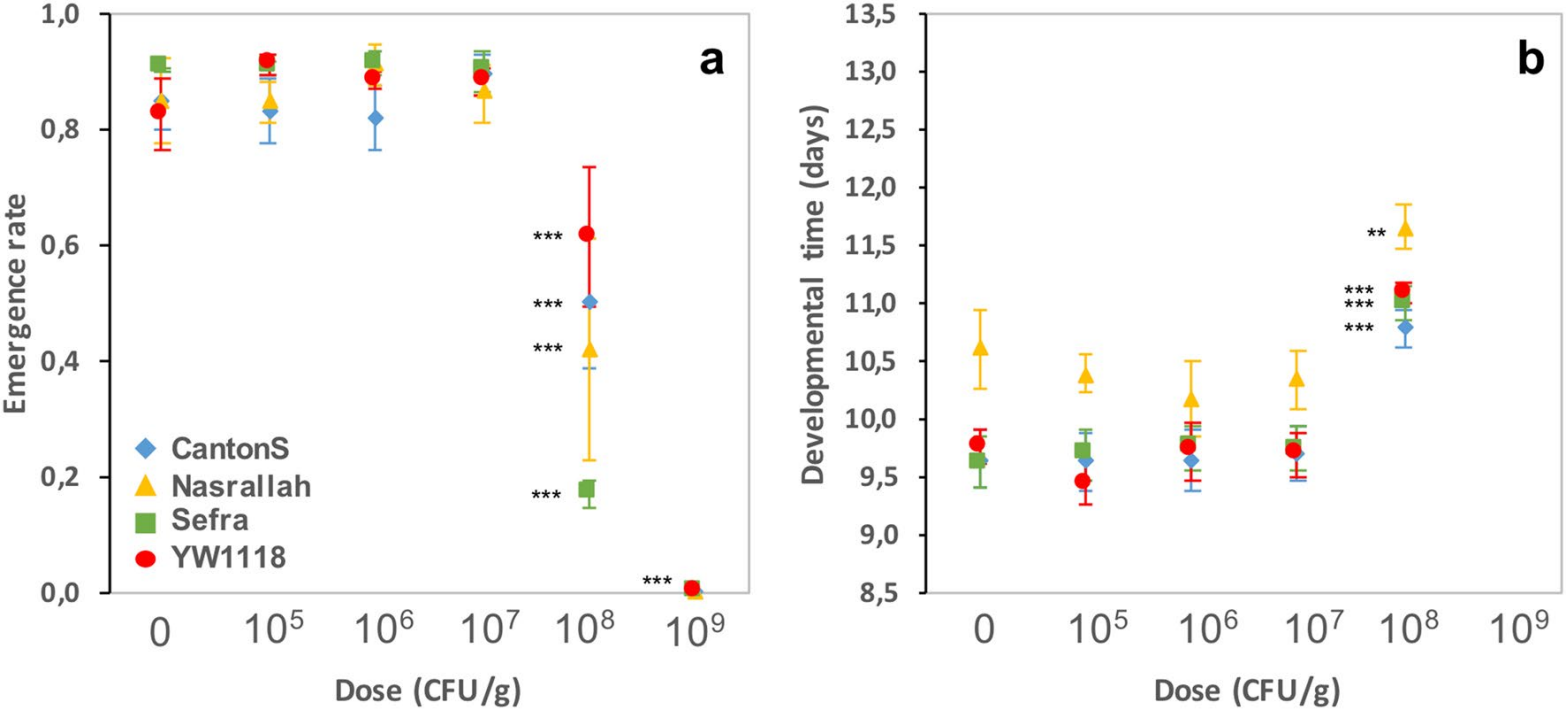
Development of four *D. melanogaster* strains on increasing doses of *Btk* **DELFIN** A. (**a**) Emergence rate and (**b**) developmental time (mean ± s.e.m.) of the strains Canton S (blue lozenges), Nasrallah (yellow triangles), Sefra (green squares), and YW1118 (red circles). *N* = 4 groups of 50 eggs per dose and fly strain for each trait. Results of post hoc comparisons of each dose to the control: **0.001 < *P* < 0.01; ***P < 0.001.

As with *D. melanogaster* Canton S, the development of three other *D. melanogaster* strains (wild-type Nasrallah and Sefra, and double mutant YW1118) was not impacted at doses up to 10^7^ CFU/g of **DELFIN** A in a high-protein/sugar-free medium. In contrast, the ER of each strain was greatly reduced and DT was increased at higher doses (Fig. 4a,b, Table 1), with no individual reaching the pupal stage at 10^9^ CFU/g (LD50 between 10^8^ and 10^9^ CFU/g). At 10^8^ CFU/g, the magnitude of effects on Canton S flies was lower than that observed on the low-protein/high-sugar medium (see Fig. 1a,b). At this dose, the ER varied between strains, the largest reduction being observed for Sefra (Table 1). We observed no dose-dependent bias in SR (Supplementary information S3).

### *Btk* formulation affects differently other *Drosophila* species

**Figure 5:**
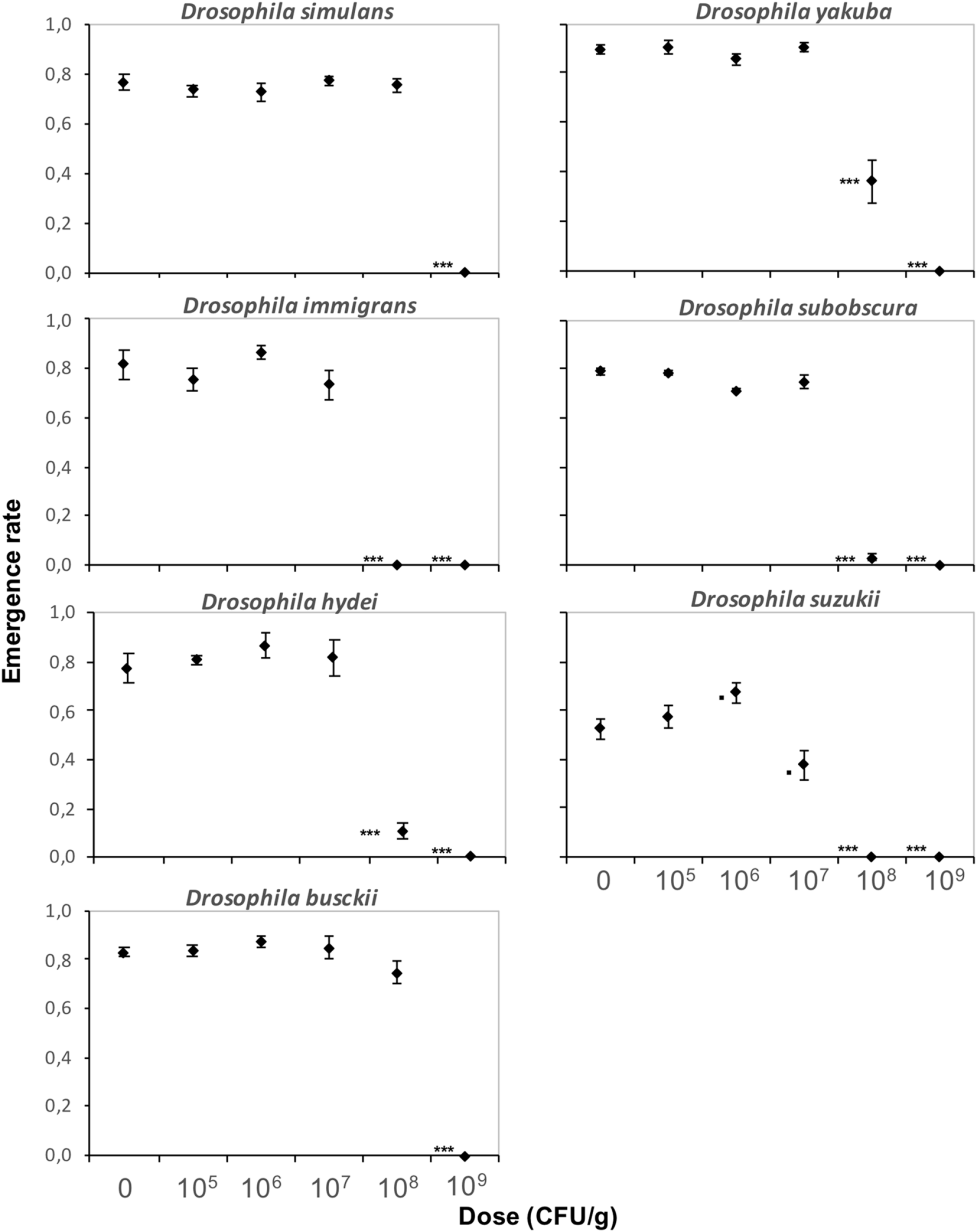
Emergence rate of seven *Drosophila* species on increasing doses of *Btk* **DELFIN** A. Mean emergence rate (± s.e.m.). *N* = 4 replicates of 50 eggs per dose for *D. simulans*, *D. yakuba*, *D. subobscura* and *D. busckii*, *N* = 4 replicates of 30 eggs per dose for *D. hydei*, *D. suzukii*, and *D. immigrans*. Results of post hoc comparisons of each dose with the control: ^•^0.05 < *P* < 0.1; ***P < 0.001.

**Figure 6:**
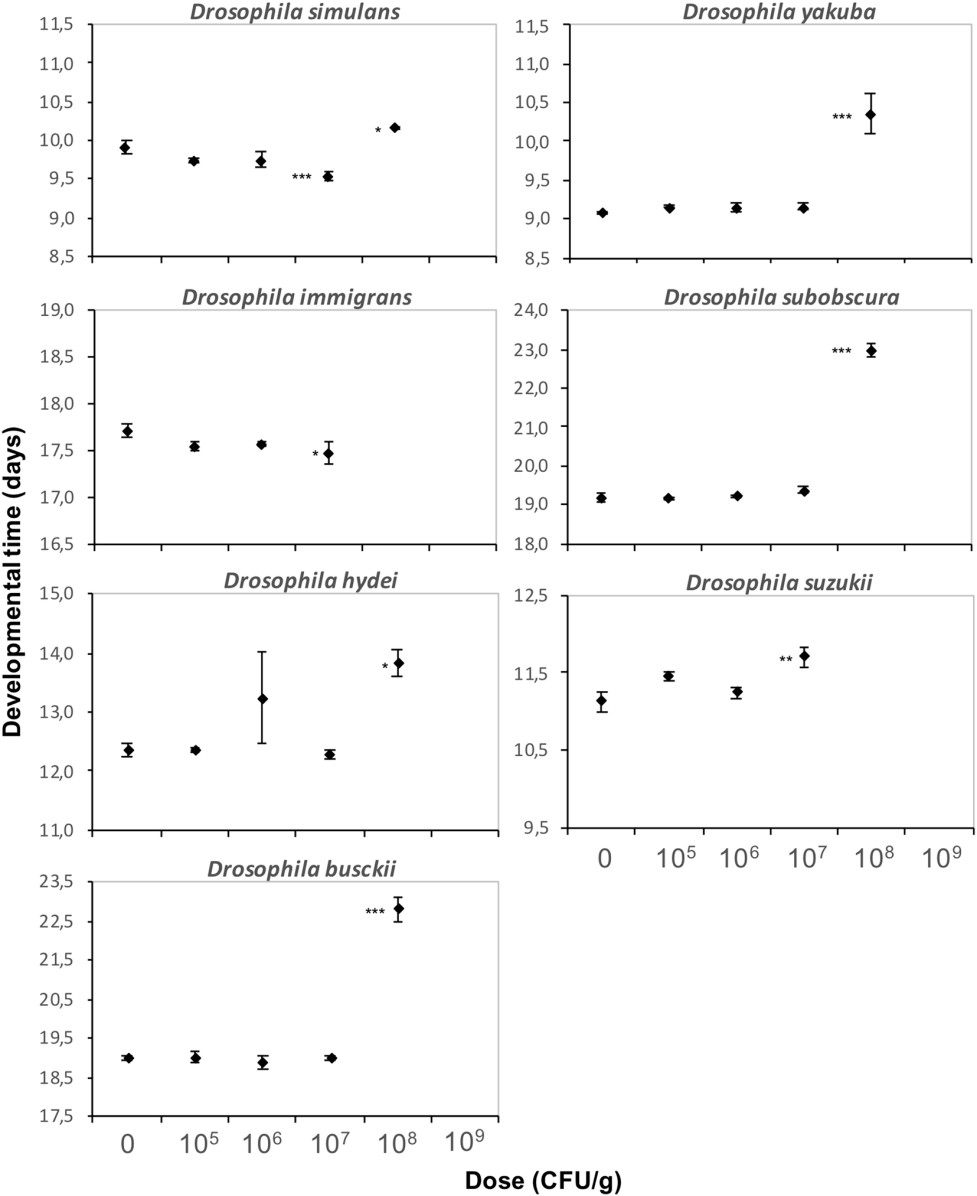
Developmental time of seven *Drosophila* species on increasing doses of *Btk* **DELFIN** A. Mean developmental time (± s.e.m.). *N* = 4 replicates of 50 eggs per dose for *D. simulans*, *D. yakuba*, *D. subobscura* and *D. busckii*, *N* = 4 replicates of 30 eggs per dose for *D. hydei*, *D. suzukii* and *D. immigrans*. Results of post hoc comparisons of each dose with the control: *0.01 < *P* < 0.05; **0.001 < *P* < 0.01; ***P < 0.001.

For seven other *Drosophila* species from different phylogenetic clades that co-occur in the field^48–51^, doses up to 10^6^ CFU/g of **DELFIN** A in a high-protein/sugar-free medium had no effect on ER and DT, whereas all individuals failed to reach the pupal stage at 10^9^ CFU/g (Figs. 5, 6). The amplitude of development alterations at 10^7^ and 10^8^ CFU/g varied among species (Figs. 5, 6; Table 1). All species were affected at 10^8^ CFU/g as was *D. melanogaster* (see Fig. 4a for comparison). *D. simulans* and *D. busckii* had unchanged ER, but DT was slightly increased for *D. simulans* (although slightly reduced at 10^7^ CFU/g; similar results with a Japanese strain, data not shown) and strongly increased for *D. busckii* (by 20%, i.e. ~ 4 days) (Figs. 5, 6, Table 1). *D. yakuba* ER and DT were similar to those of *D. melanogaster*, with an LD50 around 10^8^ CFU/g and a moderate DT increase of ~ 1 day (Figs. 5, 6, Table 1; similar results with a strain from Sweden, data not shown). The ER of *D. hydei* and *D. subobscura* were very low at 10^8^ CFU/g (LD50 below this dose), with a high DT (Figs. 5, 6; Table 1), while *D. immigrans* did not survive. No *D. suzukii* individual emerged at 10^8^ CFU/g and development was already moderately impacted at 10^7^ CFU/g (Figs. 5, 6). No dose-dependent bias in SR was detected for either species (Supplementary information S5).

### Development alterations may result from a synergy between formulation components

**Figure 7:**
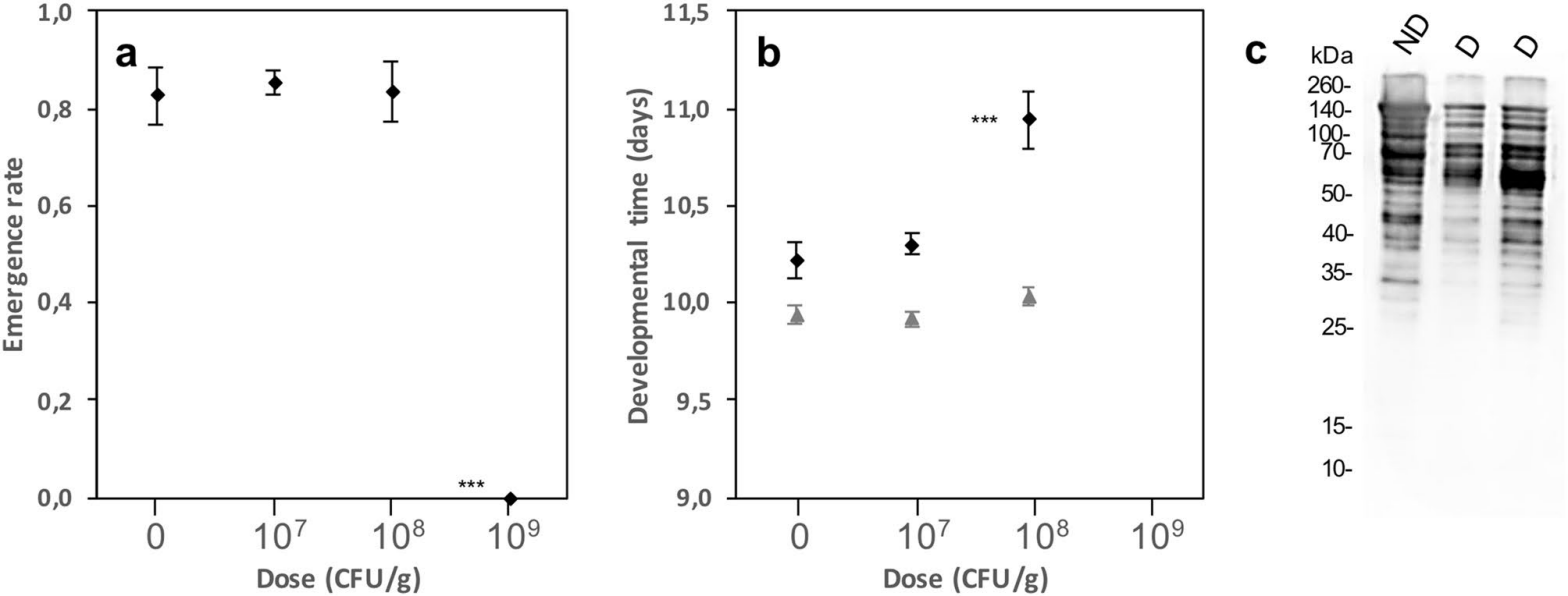
Evaluation of the role of small molecular weight components of *Btk* **DELFIN** A (dialysis; membrane cut-off: 8–10 kDa) in the altered development of *D. melanogaster* Canton S. (**a**) Emergence rate and (**b**) developmental time (mean ± s.e.m.) on increasing doses of dialyzed **DELFIN** A. *N* = 3 experiments of 4 replicates with 20 eggs per dose for the emergence rate, *N* = 2 experiments of 4 replicates per dose for the developmental time. Results of post hoc comparisons of each dose with the control: ***P < 0.001. (**c**) Anti-Cry1A probed immunoblot of non-dialyzed (ND) and dialyzed (D) suspensions showing the decrease in the amount of ~ 130/140 kDa migrating protoxins and the increase in that of the potential ~ 60/70 kDa activated toxins after dialysis.

Because some additives of commercial formulations might contribute to the observed effects, a **DELFIN** A suspension was dialyzed to remove low molecular weight additives, resuspended, and mixed with low-protein/high-sugar fly medium. At 10^7^ CFU/g, the suspension did not affect ER and DT, while no individual pupated at 10^9^ CFU/g (Fig. 7a; Table 1). At 10^8^ CFU/g, ER was not modified but DT increased in one experimental set by ~ 1 day, partially reproducing the changes observed without dialysis (Fig. 7a,b; see also Fig. 1a,b, Table 1; 3 independent experiments for ER, 2 independent experiments for DT).

The Cry1A profiles of **DELFIN** A suspensions (dialyzed or not), included a band for the 130-kDa pro-toxins and a band at 60–70 kDa likely representing the activated toxins, but also smaller fragments resulting from the degradation of Cry1A (Fig. 7c). We further explored the respective roles of *Btk* toxin fragments and spores in the alterations of *D. melanogaster* development through dialysis experiments followed by successive centrifugations to remove most of the spores and toxin crystals. Despite variation between experiments, ER was strongly affected only in one of the three experiments while DT was always significantly increased in the presence of centrifuged supernatants (Supplementary information S6). Noteworthy, the *D. melanogaster* development was not impacted in the presence of a homemade production of the *Btk* strain 4D1 containing spores, toxins, but no additives, even at the highest dose (Supplementary information S7).

## Discussion

Our study tested the side-effects of ingestion of *Bt* bioinsecticide commercial formulations (mainly made of *Bt kurstaki* strains (*Btk*) but also of *Bt israelensis* (*Bti*)) during the development of eight non-target species of *Drosophila* naturally present in treated areas. Although the recommended doses for one formulation field spray did not affect the *Drosophila* development, those 10 and 50 times higher markedly induced mortality and/or developmental delay in at least two of the species tested. We can extrapolate from our data that these doses may affect six of the eight tested species and the four strains of *D. melanogaster*. The development alterations were already strong at these doses, suggesting an occurrence from lower ingested doses but not visible in our experimental set-up. In addition, in our experimental conditions, a single *Drosophila* larva could probably not process 1 g of medium during its development. Further analyses, maybe at molecular level, would be required to determine the minimal dose affecting the fly larva. Furthermore, all the tested species except *D. simulans* were strongly affected at a 100 times the field spray dose, and no or very limited fly development occurred at the highest tested dose, equivalent to 1000 times the maximum field dose but far below the acute intoxication doses classically used in numerous studies^5^. The recommended doses for each spraying of stabilized formulation are given for a homogeneous and dry area, without overlapping. In the field, recommended repeated sprays and post-spray rainfall washouts may increase the concentration of *Bt* spores and toxins in both space and time. While a dose 1000 times the recommendations would be hardly reached in the field, the minimum doses at which the fly development was impacted and the lower doses from which developmental changes appeared could be reached. Our data also identified a first developmental window of susceptibility to *Btk* formulation during the 1st larval instar mainly explaining the adverse effects, while a second event of mortality seemed to occur at the pupation period.

In testing for generic side effects of *Bt* formulations, we observed similar patterns of developmental alterations on *D. melanogaster* but only at higher doses with two other *Btk* formulations and one *Bti* formulation (1000–2000 times the recommended spray dose). The three *Btk* formulations, based on two different bacterial strains, have similar profiles of Cry1A protoxins and activated toxins but differ in their efficient spore content. Thus, the type of formulation and probably the additives, may explain the observed variation in the dose effect.

The impacts of *Btk* formulations on the development of *D. melanogaster* are consistent with growing evidence suggesting partial specific targeting of *Bt*^12,26,27^.The consensus on the mode of action of *Bt* after ingestion by insects relied until recently on the key steps of the specific binding of proteolyzed *Bt* toxins to midgut epithelial cell receptors, defining targets for each *Bt* subspecies^12,15,17^. Several primary and secondary types of toxin receptors have been identified in the Lepidoptera and Diptera mosquitoes such as cadherin-like proteins, aminopeptidases, GPI-anchored alkaline phosphatases^8^, and more recently the ATP dependent binding cassette reporter C2^52^. No orthologues of the Lepidoptera cadherin-like Cry receptors were found in *Drosophila*^52^*,* supporting the idea of the lack of effect of *Btk* toxins on these flies. Yet, *Drosophila* flies may have other types of Cry receptors, therefore explaining the developmental impacts observed, but this remains to be investigated. In addition, the possible lack of solubilization of the protoxin crystals and of proteolytic activation of toxins by proteases in the fly gut, both required for Cry activity in insects’ larvae^15^, would be possibly compensated by the substantial amounts of active Cry1A toxin fragments in *Btk* formulations. Other *Btk*-synthesized toxins present in the formulations could also be players in the observed cross-order activity since some, like Cry2A, have an insecticidal effect on both Lepidoptera and Diptera^53^.

Since ingestion of *Bacillus subtilis* or *Btk* Cry-free does not affect the development of *D. melanogaster*, the observed development alterations cannot result solely from a severe disturbance of digestion and nutrient absorption/competition due to the presence of high loads of spores/bacteria in the larval gut throughout development. This suggests a synergistic action of *Btk* spores and Cry toxins, consistent with the *Bt* action models on insect larvae, i.e. the breach of the intestinal epithelium allowing colonization of the hemocoele by the gut bacteria, including *Bt* spores^15,17,18^. The partial mimicry of mortality rates and developmental delays in preliminary dialysis assays would also support a contribution of diffusible low molecular weight compounds in *Btk* formulations (e.g. residues of culture media, salts, additives) to these development alterations. Furthermore, there is no impact on the development of *D. melanogaster* of the ingestion of homemade spores and Cry toxins of the *Btk* strain 4D1 used without additives even at the highest dose (or HD1, a reference strain used also as a control). Unlike commercial *Btk* formulations, *Btk* 4D1 culture contains few activated Cry toxins and smaller toxin fragments, advocating the possible contribution of such fragments to the cross-order activity of *Btk* formulations on *Drosophila*. Completion of these preliminary tests is required to further investigate the mechanisms of the harmful effects of *Btk* formulations on the development of *Drosophila* and unravel the respective roles of the synergy spores/toxins/crystals and of formulation additives.

As reported for *D. suzukii* exposed to *Btk* cultures^45^, *D. melanogaster* mortality on the *Btk* formulation occurred mainly during early development. Only ∼40% of the 1st and 2nd instar larvae died at the highest dose tested (Fig. 2) while no individual reached the pupal stage, the remaining mortality likely occurring during, or at the end of, the 3rd larval instar, possibly due to the delayed action of the gut accumulated *Btk* spores and toxins at the onset of pupation. Interestingly, developmental alterations (mortality, delayed emergence) mimicked those typically caused by nutritional stress in insect larvae^54,55^. Accordingly, the developmental alterations were partially rescued on a protein rich fly medium, probably by a compensatory protein intake, as in other arthropod species^55–57^. Also, the sex ratio of flies was strongly biased towards males after development on the *Btk* formulation dose affecting fly emergence and under protein restriction. This highlights the importance of nutritional conditions such as protein restriction, added to sex-specific differences in larval susceptibility to environmental stressors, here the accumulation of *Btk* formulation, as already reported previously in *D. melanogaster*^58^.

The development on sublethal doses of *Btk* formulation did not dramatically affect the longevity of *D. melanogaster* adults, nor their lifetime offspring number. Exposure during development to doses of *Btk* formulation that slightly and strongly reduced the likelihood of reaching the adult stage even provided a dose-dependent longevity benefit to the surviving flies and tended to increase their offspring number. Exposure to the *Btk* formulation throughout development probably selected resistant and/or tolerant individuals, reminding the increased longevity of adult insects having survived a nutritional stress during development^59,60^, or withstood environmental stressors^61^.

The origin of *Drosophila* (species, population/strain) influenced the amplitude of the development alterations induced by the *Btk* formulation. For *D. melanogaster*, all the tested strains were equally susceptible, but with variation in the dose effect amplitudes. These differences in susceptibility suggest a possible spatial and temporal heterogeneity of the potential impacts of *Btk* spraying among natural *D. melanogaster* populations. Among the other seven species tested, differences occurred in the susceptibility to the *Btk* formulation, in terms of nature of development alterations and effect amplitudes, regardless of their phylogenetic distances. For the subgenus *Drosophila*, *D. simulans* was less sensitive than its sister species *D. melanogaster*, while the African *D. yakuba* experienced similar development impacts as *D. melanogaster*. Although phylogenetically close, *D. melanogaster* and *D. simulans* would respond very differently to *Btk* formulations, with a possible advantage for *D. simulans* in case of competition. *D. immigrans*, *D. subobscura* and *D. hydei* were similarly more sensitive than *D. melanogaster*. The phylogenetically distant *D. busckii* (subgenus *Dorsilopha*) was the least affected of all the species in terms of developmental mortality, but its development was strongly delayed. The species *D. melanogaster*, *D. simulans*, *D. hydei*, *D. immigrans*, and *D. busckii* belong to the guild of cosmopolitan domestic *Drosophila* species, *D. subobscura* is a sub-cosmopolitan species, and *D. busckii* is an opportunistic frugivorous species^62,63,64^. They all coexist frequently and compete on the same discrete and ephemeral rotting fruit patches, with seasonal variations in the composition of the fly community^47–49,62^. Differences in species susceptibility to *Btk* formulations could modify the conditions of larval competition, therefore adding local and temporal variations in the composition of *Drosophila* communities. The potential side-effects of *Bt* sprays on non-target *Drosophila* communities would be hardly predictable as they would depend on spatial patterns of *Bt* accumulation. A formal mesocosm study of *Drosophila* community dynamics under exposure to *Btk* formulation, at least under semi-field conditions, would help to identify the consequences of *Bt* accumulation on species competition and community composition. The exposure to *Btk* formulation also impacted the development of the invasive *D. suzukii*, as recently reported^45^, this species being the most susceptible with effects already clearly detectable at only 10 times the recommended spray dose*.* Compared to the other tested species living on rotten fruits, *D. suzukii* threatens fruit production since it feeds and lay eggs inside healthy ripening soft fruits^63–65^, colonizing orchards and vineyards earlier during the fruit season. The higher susceptibility of *D. suzukii* to the accumulation of *Btk* formulation in the environment might reduce the possible ecological burden of its invasion for local communities of fruit-eating *Drosophila* in orchards. Alternatively, since *D. suzukii* attacks on fruits can accelerate their decomposition, its increased susceptibility may reduce the number of fruits available for the rotting fruit-eating *Drosophila* species.

Overall, our data show that the ingestion of *Btk* bioinsecticides above the recommended spray doses can potentially impact non-target *Drosophila* flies, with an effect amplitude depending on both the formulation and the fly species. Although our study was carried out under controlled laboratory conditions, which may considerably differ from those of the field (e.g. temperature, pH, humidity, food availability, presence of predators/parasites/pathogens, etc.…), standard laboratory strains and flies derived from recently collected populations exhibited similar patterns of developmental alterations, suggesting our results are likely generalizable. Recent studies have reported similar adverse side-effects due to repeated spraying of the *Bti* formulation on non-target organisms^25^, and indirectly on predators via food webs^66^. From these studies and our data here, care should clearly be taken when using *Bt* bioinsecticides to avoid, or at least minimize, potential side-effects on non-target organisms and therefore on biodiversity. At last, *D. melanogaster* could serve as a model species to identify the mechanisms underlying these side effects and/or the potential emergence of resistance to these bioinsecticides.

## Methods

### Commercial formulations, *Bacillus* productions and Colony Forming Unit

*Btk* brands (serotype 3a, b, c^67^) were **DELFIN** (two formulations named A and B; strain SA-11; wettable granules, Valent BioSciences, AMM 9200482, 32,000 UI/mg) and **Scutello DF** (a Dipel sub-brand; strain ABTS-351; wettable granules, Biobest, AMM 2010513, 540 g/kg). *Bti* brand (strain HD-14; serotype 14^67^) was **VectoBac WG** (wettable granules, Bayer, AMM 2020029, 3000 UTI/mg). For each formulation, the number of viable spores was estimated by counting Colony Forming Units (CFUs) developing on LB agar after overnight incubation at 30 °C from serial dilutions of a suspension (Colony Forming Units (CFU) per mg of product). Estimations were 5 × 10^7^ CFU/mg for **DELFIN** A; 2.5 × 10^7^ CFU/mg for **DELFIN** B; 2.2 × 10^7^ CFU/mg for **Scutello DF**; 6 × 10^7^ CFU/mg for **VectoBac WG**, and were stable during the experiments time frame. Our CFU estimations agree with those indicated for the formulations, between 1–5 × 10^13^ CFU/kg. Manufacturer-recommended **DELFIN** doses for one spraying range from 0.15 to 1.5 kg/ha depending on the crop. Based on our CFU estimations, this corresponds to 7.5 × 10^4^ to 7.5 × 10^5^ CFU/cm^2^ of **DELFIN** A, and 3.75 × 10^4^ to 3.75 × 10^5^ CFU/cm^2^ of **DELFIN** B. **Scutello DF** is used at 0.1 to 1 kg/ha, equivalent to 2.2 × 10^4^ to 2.2 × 10^5^ CFU/cm^2^. **VectoBac WG** is used at 0.125 to 1 kg/ha, equivalent to 7.5 × 10^4^ to 6 × 10^5^ CFU/cm^2^.

The acrystalliferous *Btk* 4D22 strain (depleted for the Cry toxin-encoding plasmids^68^; Bacillus Genetic Stock Center, https://bgsc.org, Columbus, USA), and a *Drosophila* non-pathogenic *Bacillus subtilis* (from Dr. E. Bremer, University of Marburg, Germany) were grown at 30 °C in the sporulation-specific medium (Bactopeptone 7.5 g, KH_2_PO_4_ 3.4 g, K_2_HPO_4_ 4.35 g, glucose 7.5 g, PGSM salts (MgSO_4_·7H_2_O, MnSO_4_·H_2_O, ZnSO_4_·7H_2_O, FeSO_4_·7H_2_O) 5 mL, CaCl_2_ 0.25 M, distilled water qsp 1L, pH 7.2) until sporulation (about 14 days). Vegetative cells were eliminated (1 h at 70 °C) and after centrifugation (4500 rpm, 20 min, 4 °C), a spore pellet was collected, washed with sterile water, and lyophilized. Production CFUs was estimated as described above.

### Fly stocks

*Drosophila melanogaster* strains (phylogenetic subgroup: melanogaster) were wild-type Canton S (Bloomington Drosophila Centre) used as a reference strain, “Nasrallah” from Tunisia (strain 1333, Gif-sur-Yvette), a French field-collected strain “Sefra” (Southern France, 2013), and the double mutant standard strain YW1118 (white and yellow mutations; gift from Dr. B. Charroux, IBD, Marseille-Luminy). Other *Drosophila* species were *D. simulans* (Gif strain 1132; phylogenetic subgroup: melanogaster), *D. yakuba* (Gif strain 1880; phylogenetic subgroup: melanogaster), *D. hydei* (phylogenetic subgroup: hydei) and the invasive *D. suzukii* (phylogenetic subgroup: immigrans) (both kindly provided by Dr. R. Allemand, LBBE, University Lyon 1), *D. immigrans* (phylogenetic subgroup: immigrans), *D. subobscura* (phylogenetic subgroup: obscura), and *D. busckii* (*Dorsilopha* subgenus). The populations of the last three species were initiated from individuals collected in South-East of France in Spring 2015.

All the flies were maintained at controlled densities (150–200 eggs/40 ml of fly medium) under standard laboratory conditions (25 °C, or 20 °C for recently collected species, 60% relative humidity, 12:12 light/dark cycle), on a high-protein/sugar-free fly medium (10% cornmeal, 10% yeast, 0% sugar). In our laboratory, the *D. melanogaster* Canton S was also reared on a standard low-protein/high-sugar medium (8% cornmeal, 2% yeast, 2.5% sugar). For each experiment, eggs, larvae and flies were maintained under standard conditions.

### General method of intoxication and dose–response assay

For the dose–response assays, formulations and spore productions were serially diluted in buffer, and 100 µl of each dilution was homogenized thoroughly with 1 g of fly medium (100 µl/g doses). Eggs and defined larval instar were collected from stock vials and transferred to the intoxication vials and dishes as described below. They were then reared until the fly emergence, and, for larval susceptibility tests, until the desired development stage was reached, or for 24 h for early larvae of the 1st and 2nd instars. Equivalent control groups were transferred on fly medium homogenized with the same volume of buffer alone.

### Development-related traits and larval survival

Developmental traits upon intoxication throughout the entire development of the *D. melanogaster* strains and the other *Drosophila* species were evaluated on a precise number of viable eggs collected from mass oviposition and transferred to intoxication vials containing fly medium (high sugar/low protein or high protein/sugar free as indicated) mixed with formulations or spore productions at doses ranging from 1 × 10^5^ or 5 × 10^5^ CFU/g (mean equivalent to the manufacturer recommendations; Supplementary information S1) to 10^9^ CFU/g. Eggs were let to develop until fly emergence. Egg density was set at 8–10 eggs/g of medium (10 eggs/g on 2 g medium in small vials Ø 3.3 cm, surface ~ 8.5 cm^2^, 0.24 g/cm^2^ for tests on *D. melanogaster* Canton S; 8 eggs/g on 6 g medium in large vials Ø 4.6 cm, surface ~ 16 cm^2^, 0.37 g/cm^2^ for strains and species comparisons), except for *D. hydei*, *D. suzukii* and *D. immigrans* for which the egg density was reduced by half because of their reproductive biology (5 eggs/g on 6 g). Numbers and sex of emerging flies were recorded once a day until the day on which the first pupae of the next generation forms. The emergence rate (proportion of flies emerged from the initial eggs), the developmental time (mean number of days for development completion) and the sex-ratio (proportion of male flies) were calculated for each replicate vial.

For the larval susceptibility tests, survival was measured on 20 eggs/larvae of *D. melanogaster* Canton S at a suitable instar collected from a 4-h mass oviposition and transferred to small dishes (Ø 3 cm, surface ~ 7 cm^2^) containing 1 g of high-protein/sugar-free fly medium (less limiting for early larval development) homogenized with **DELFIN** A doses ranging from 10^5^ CFU/g to 10^9^ CFU/g. First and 2nd instar larvae were used since growth is exponential during these two instars^39,69^ and larvae are more likely to be heavily exposed to the bioinsecticide. Proportion of surviving larvae was measured at the indicated developmental stage for the cumulative survival test, or 24 h later. For cumulative survival, unhatched eggs were discarded from the counting. The pH of the fly medium was measured for the *Btk* dose-responses; neither the presence of *Btk* formulation nor the dose altered it (Supplementary Information S4).

### Adult fitness-related traits

For longevity and total offspring number measurements, *D. melanogaster* Canton S eggs from mass oviposition were transferred to several vials with low-protein/high-sugar medium mixed with 5 × 10^6^, 5 × 10^7^ or 10^8^ CFU/g of **DELFIN** A. For each dose, flies from the vials were pooled 2 days after emergence and groups of 15 males and 15 females were transferred on the same medium without **DELFIN** A. Flies were transferred to a new vial every 3–4 days and previous vials were incubated for the offspring to develop. Mortality and sex of dead flies were recorded daily until the last fly died. Offspring numbers were counted from the first emergence until pupae of the next generation appeared. Two experimental blocks were set. Due to differences in the duration of the experiment, the offspring numbers of all vials of each dose of **DELFIN** A were summed for each experimental block.

### Dialysis and cry toxin analysis

For some products, additives can be more harmful than the active ingredient^70^. To eliminate low molecular weight additives present in the formulation, a **DELFIN** A suspension at 2 × 10^10^ CFU was dialyzed against PBS (KH_2_PO_4_ 1 mM, Na_2_HPO_4_(2H_2_O) 3 mM, NaCl 154 mM, pH 7.2), overnight at 4 °C, using an 8–10 kDa cut-off membrane (ZelluTrans, Roth). CFUs of the dialyzed suspension were estimated as above. The effects on the emergence rate (ER) and developmental time (DT) of *D. melanogaster* Canton S were analysed on 20 eggs, at 10^7^, 10^8^ and 10^9^ CFU/g of dialyzed and also centrifuged suspension mixed with 2 g of low-protein/high-sugar fly medium. The dialyzed suspension was subject to a 12.5% SDS-PAGE and compared to the non-dialyzed suspension after silver staining. The presence of Cry1A pro-toxins, activated toxins and toxin fragments was probed by Western-blot using an in-house anti-Cry1A rabbit polyclonal antibody.

### Data analysis

Data were analysed with mixed-effects models with replicates as random effects. Dose of *Btk* formulation/spore production, *D. melanogaster* strain, *Drosophila* species or developmental stage, experimental block where necessary, and appropriate 2-way interactions between these factors, were included as fixed effects. Main fixed effects and their interactions were tested with log-likelihood ratio tests. Post hoc pairwise comparisons were made for *D. melanogaster* strains, formulation/spore treatments, and between the control and the other doses.

Data of emergence rate, sex-ratio, and larval survival were analysed with generalized linear models with binomial distribution and logit link; for emergence rate data, the model was also bias-corrected to correct for multiple 0 values, and with replicate as random effect. To run the *post-hoc* analysis, the same model including replicates as fixed effect was applied to the data of emergence rate and provided similar results. Developmental time (1/x transformed) and offspring number were analysed with linear models. Adult longevity data were analysed with proportional hazard Cox regression models, including fly sex and formulation dose as fixed effects, and replicate as a random effect. Analyses were performed in R^71^ using the packages lme4^72^, brglm^73^, multcomp^74^, survival^75^, and coxme^76^.

## Supplementary information

Supplementary Information.
